# An Unusual Case of Overlapping Immunoglobulin G4-Related Disease and Systemic Lupus Erythematosus

**DOI:** 10.7759/cureus.75362

**Published:** 2024-12-09

**Authors:** Pilar Burillo Simões, João Martins, Maria Do Mar Menezes, João Sousa, Cristina Jorge

**Affiliations:** 1 Nephrology, Unidade Local de Saúde de São José, Lisbon, PRT

**Keywords:** autoimmune overlap syndrome, igg4-related kidney disease, immunosuppression therapy, kidney biopsy, systemic lupus erythematosus disease

## Abstract

Immunoglobulin G4-related disease (IgG4-RD) and systemic lupus erythematosus (SLE) are multisystemic autoimmune disorders that can present with renal manifestations. Overlapping cases of these diseases are extremely rare and present both diagnostic and therapeutic challenges. We report the case of a 70-year-old male with a history of autoimmune pancreatitis, who was admitted with fatigue, weight loss, and worsening kidney function. Laboratory tests revealed anemia with a positive Coombs test, leucopenia, elevated IgG4, hypocomplementemia, and positive results for ANA, anti-double-stranded DNA (dsDNA), anti-nucleosome, anti-RP11 antibodies, and rheumatoid factor. A spot urine sample showed subnephrotic proteinuria without hematuria. The patient met the criteria for both SLE and possible IgG4-RD, but the cause of the worsening renal function remained unclear, prompting a kidney biopsy. The biopsy revealed a lymphoplasmacytic infiltrate, storiform fibrosis, and IgG4-positive staining, consistent with IgG4-related tubulointerstitial nephritis, but without evidence of lupus nephritis. The patient was treated with prednisolone, resulting in improvement of both his symptoms and kidney function. However, significant leukopenia, anemia, and elevated anti-dsDNA titers persisted, which were presumed to be secondary to the overlapping SLE. Hydroxychloroquine and azathioprine were added to the treatment regimen, leading to improvement in cytopenias at the three-month follow-up. This case underscores the importance of kidney biopsy in suspected overlapping autoimmune diseases for identifying kidney involvement and guiding treatment, although evidence regarding optimal therapy remains limited.

## Introduction

Autoimmune kidney diseases encompass a wide range of conditions with overlapping clinical and laboratory findings, making differential diagnosis particularly challenging [1,2]. Both immunoglobulin G4-related disease (IgG4-RD) and systemic lupus erythematosus (SLE) are multisystemic immune-mediated diseases that can present with renal involvement [3].

IgG4-RD is more common in middle-aged or older men and is characterized by a hallmark plasmacytic-rich fibroinflammatory infiltrate, typically affecting the pancreas and other exocrine glands. Renal involvement is rare, most often manifesting as tubulointerstitial nephritis. The role of IgG4 in the pathogenesis of the disease - whether active or bystander - remains unclear [4].

SLE predominantly affects women of childbearing age and frequently presents with joint, skin, renal, or hematologic manifestations, as well as prominent immunologic abnormalities such as positive antinuclear antibodies (ANA). Lupus nephritis occurs in approximately 50% of cases, with nearly universal glomerular involvement [3].

The European Alliance of Associations for Rheumatology and the American College of Rheumatology have published joint classification criteria for IgG4-RD, which include clinical, radiological, and pathological findings of specific organ involvement, along with exclusion criteria for other diseases, including SLE [5]. Overlapping cases of IgG4-RD and SLE are rare and present unique diagnostic and therapeutic challenges due to the lack of evidence regarding optimal management strategies.

## Case presentation

A 70-year-old white male was admitted to the nephrology ward due to fatigue, unquantified weight loss, and acute kidney injury. His medical history included hypertension and cerebrovascular disease.

Five years earlier, he had been diagnosed with possible IgG4-RD. He initially presented with jaundice and abdominal pain. Subsequent investigations revealed cholestasis, mild kidney dysfunction (creatinine 1.3 mg/dL, urea 104 mg/dL), and elevated IgG4 (231 mg/dL, reference value <125 mg/dL). Abdominal MRI showed an enlarged pancreas with diffusion restriction, and aspiration cytology revealed typical lymphoplasmacytic infiltrate with minimal IgG4 expression. He was started on prednisolone (0.5 mg/kg) for eight weeks, leading to resolution of symptoms and normalization of creatinine and IgG4 levels. No further tests were performed at that time.

At present, blood work revealed anemia, leukopenia, worsening kidney function, and subnephrotic proteinuria without erythrocyturia. Immunological studies showed elevated IgG4, low C3 and C4, and positive results for several antibodies, including anti-nuclear, anti-double-stranded DNA (dsDNA), anti-nucleosome, anti-RP11 antibodies, and rheumatoid factor. Detailed test results are shown in Table 1.

**Table 1:** Blood and urine test results at the time of hospital admission ANA, antinuclear antibodies; IgG4-RD, immunoglobulin G4-related disease

Test	Result	Normal range
Hemoglobin (g/dl)	6.1	13.0-17.0
Erythrocytes (/uL)	2,100	4,400-5,900
Reticulocytes (/uL)/adjusted reticulocyte index (%)	52,900/0.48	25,000-100,000/0.5-1.5
Leukocytes (/uL)	1,750	4,500-11,000
Neutrophils (/uL)	580	2,000-8,500
Eosinophils (/uL)	20	0-600
Basophils (/uL)	0	0-100
Lymphocytes (/uL)	870	900-3,500
Monocytes (/uL)	280	200-1,000
Platelets (/uL)	193,000	150,000-450,000
Direct Coombs test	Weak positive	-
Ferritin (ng/ml)	449	30-400
Transferrin saturation (%)	30.5	20-40
Folic acid (ng/ml)	4.9	3.1-17.5
Vitamin B12 (pg/ml)	701	197-771
Creatinine (mg/dL)	4.06	0.67-1.17
Urea (mg/dL)	145	16.6-48.5
Amylase (U/L)	123	13.0-53.0
Lipase (U/L)	103	13.0-60.0
C3 (g/L)	0.45	0.9-1.8
C4 (g/L)	0.04	0.1-0.4
Electrophoresis		
Total proteins (g/L)	81.6	60.0-83.0
Albumin	33.9	36.0-55,0.
Alpha-1 globulins	3.2	1.8-41
Alpha-2 globulins	6.7	4.5-9.8
Beta-1 globulins	3.3	3.0-6.0
Beta-2 globulins	4.1	2.0-5.4
Gama globulins	30.5	7.1-15.6
Immunoglobulin G (g/L)	35.3	7.0-16.0
IgG1 (mg/dL)	2,280	422-1,290
IgG2 (mg/dL)	535	117-747
IgG3 (mg/dL)	399	40-130
IgG4 (mg/dL)	393	1-291
ANA	0.486111	-
Anti-dsDNA antibodies (IU/mL)	377	<40
Anti-nucleosome antibodies (IU/mL)	161	<20
Anti-RP11 antibodies	2+	-
Anti-SSA antibodies	Negative	-
Anti-SSB antibodies	Negative	-
Rheumatoid factor (IU/mL)	97.6	<15
Urinalysis	Albumin 15 mg/dl, without erythrocyturia	-
Protein-to-creatinine ratio in urine (mg/g)	490	<200

**Figure 1:**
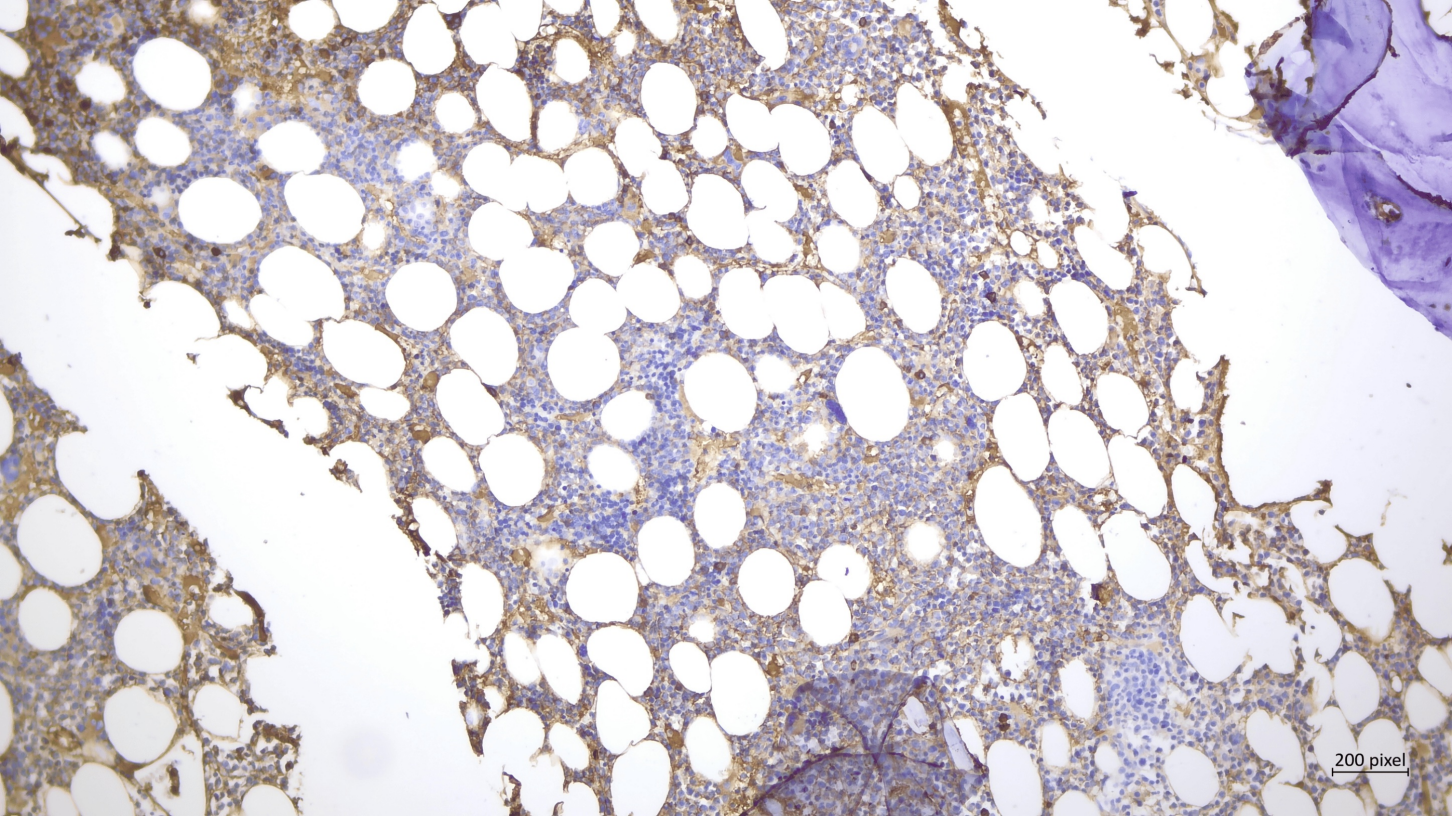
Bone marrow biopsy Plasmacytic infiltration of the bone marrow, with minimal staining for IgG4. IgG4-RD, immunoglobulin G4-related disease

A bone marrow biopsy was performed to investigate the causes of low-reticulocyte anemia and leukopenia and to exclude bone marrow involvement in IgG4-RD. The biopsy revealed a reactive pattern with increased cellularity and interstitial plasmacytic infiltrate, with less than 10% of the plasmocyte population positive for IgG4 and no signs of obliterative phlebitis (Figure 1).

**Figure 2:**
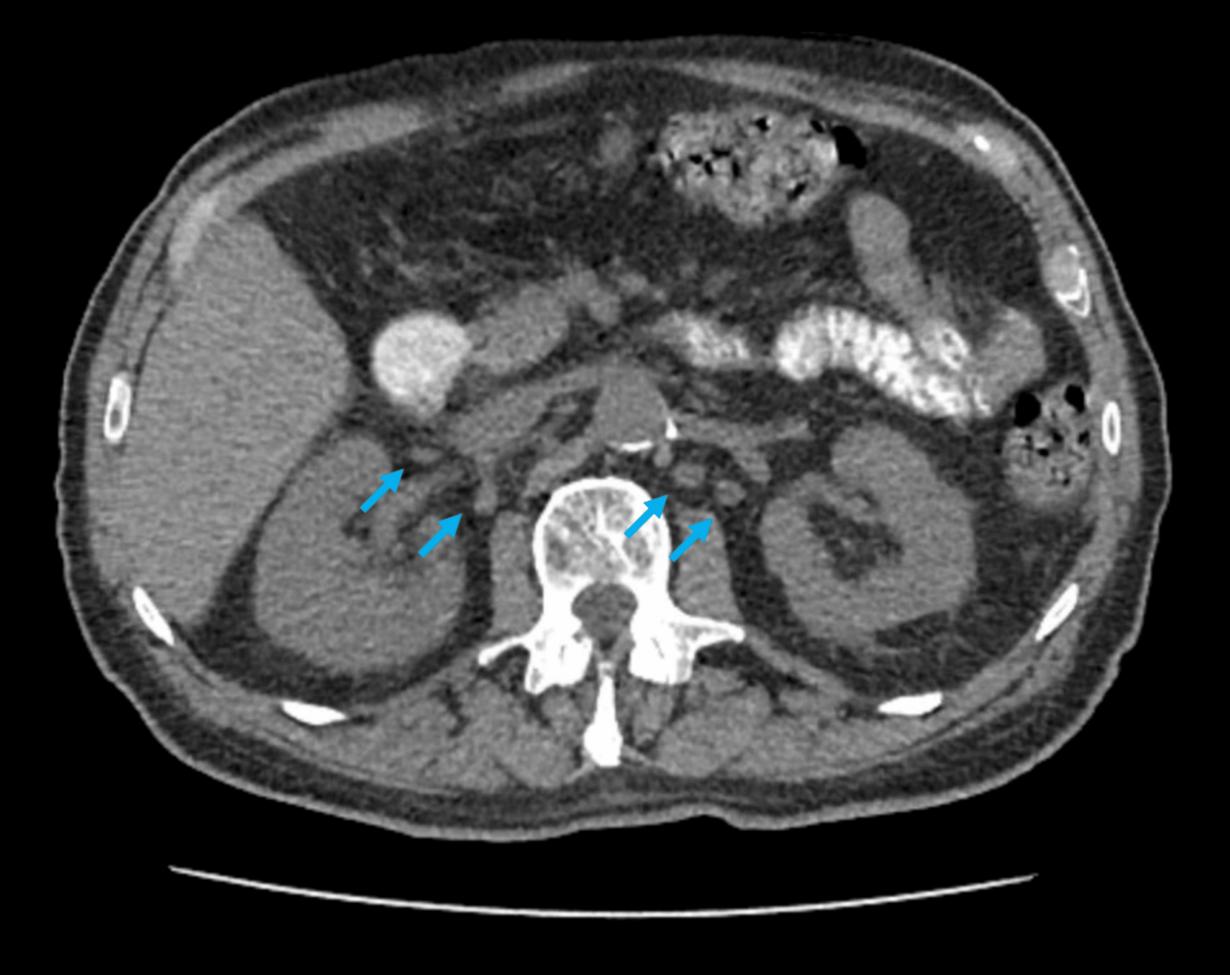
Abdominal CT CT scan showing multiple abdominal lymphadenopathies (arrows).

An abdominal CT scan was performed, showing diffuse lymphadenopathy throughout all thoracic and abdominal chains, without retroperitoneal fibrosis or specific organ involvement (Figure 2). Due to the patient’s worsening kidney function, we opted for a non-contrasted exam despite its lower sensitivity, as MRI was unavailable at that time.

Raised IgG4 levels and imaging findings, along with the patient’s previous history, supported the diagnosis of IgG4-RD. However, the presence of ANA antibodies, specifically against dsDNA, along with direct Coombs test positivity in the absence of hemolysis, met the immunological criteria for SLE. Additionally, both renal and hematological involvement remained unclear, despite bone marrow biopsy ruling out significant IgG4-positive plasmocyte infiltration.

**Figure 3:**
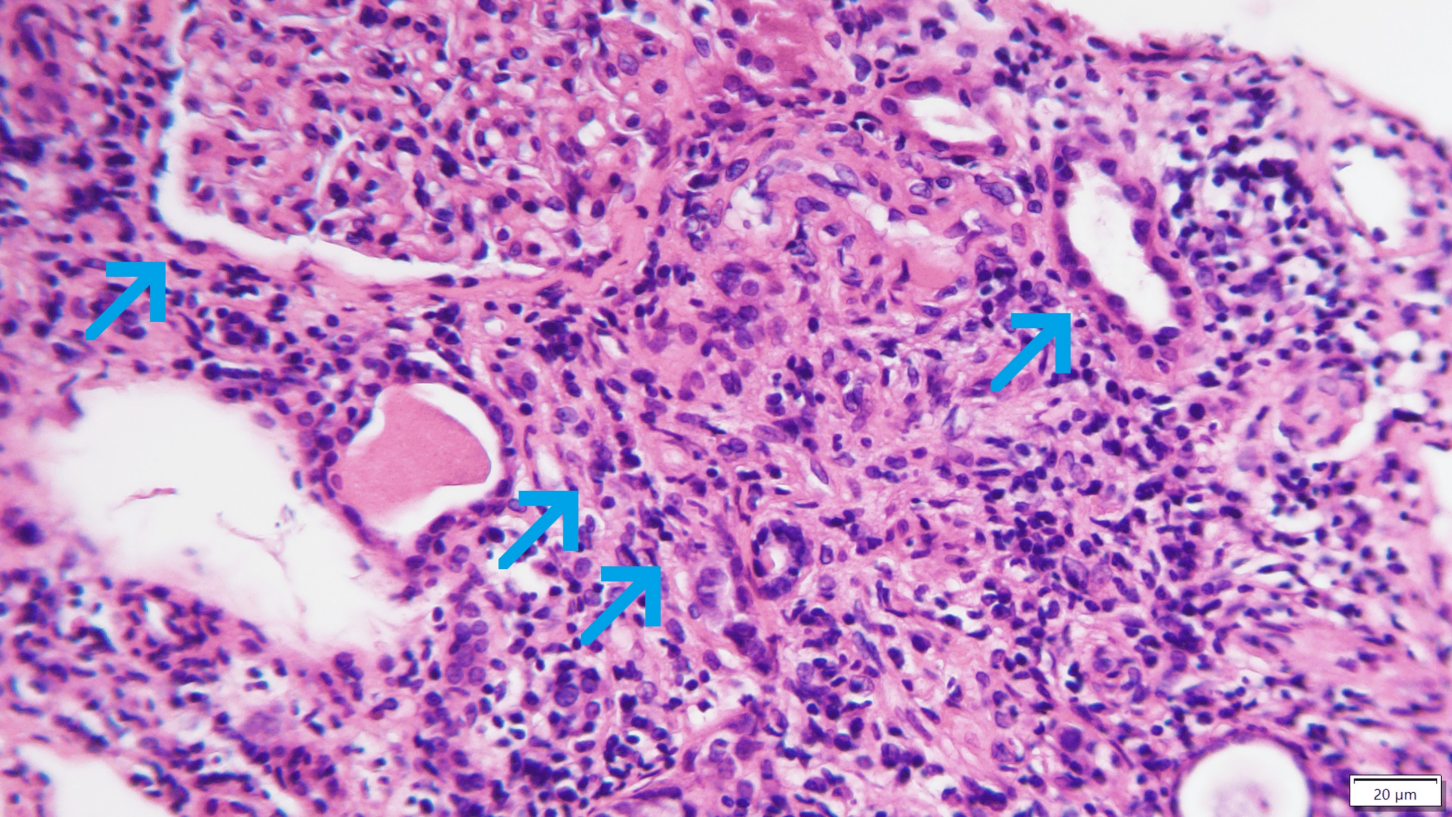
Kidney biopsy Kidney biopsy section (periodic acid-Schiff staining). The left arrow points to a glomerulus, and the right arrow points to a tubule. Arrows in the center highlight the renal interstitium, which exhibits a characteristic storiform fibrosis pattern of IgG4-related disease. The light pink connective tissue is arranged in bands forming a whorled pattern. IgG4-RD, immunoglobulin G4-related disease

These findings prompted the decision to perform a kidney biopsy, especially after an ultrasound revealed normal-sized kidneys (125 and 130 mm in diameter) with reasonable cortical thickness and corticomedullary differentiation. The kidney biopsy showed lymphoplasmacytic infiltrate (70%), extensive storiform fibrosis, and IgG4 staining (>10 cells per high-power field), with preserved glomeruli and vessels (Figure 3).

The diagnosis of interstitial nephritis due to IgG4-RD was made, and the patient was started on prednisolone (0.6 mg/kg). Kidney function and blood cell counts improved rapidly, and the patient was discharged ten days later. Considering a mixed etiology for the persistent anemia, with probable chronic kidney disease contribution, the patient was also started on the erythropoiesis-stimulating agent (ESA) darbepoetin at 20 µg/week.

Steroid therapy was gradually tapered and completely stopped after two months. At the first follow-up visit (immediately after steroid cessation), kidney function remained stable (serum creatinine 2.2 mg/dL) and IgG4 levels were normal, but hematologic abnormalities persisted and worsened despite increasing darbepoetin to 60 µg/week. Anti-dsDNA antibodies remained positive, and C3 had decreased between visits. Based on the 2012 Systemic Lupus Erythematosus International Collaborating Clinics (SLICC) criteria, the patient met both clinical (anemia and leukopenia) and immunological (positive ANA, dsDNA, low complement, and positive direct Coombs test) criteria, supporting the diagnosis of SLE.

Given the overlap with SLE, the patient was restarted on prednisolone, and hydroxychloroquine (400 mg) and azathioprine (25 mg) were added to the therapeutic regimen. At the second follow-up visit, two months later, hemoglobin levels remained stable without the need for an increased ESA dose, and dsDNA levels were nearly normal. Leukocyte count increased, but this is a known effect of steroid therapy. Additional follow-up will be needed to determine if this represents sustained improvement. A summary of blood test changes is shown in Table 2. The patient is currently still on steroid weaning with close monitoring.

**Table 2:** Blood and urine test results, along with therapeutic changes during follow-up dsDNA, double-stranded DNA; IgG4-RD, immunoglobulin G4-related disease

Test	At discharge	Follow-up visit 1	Follow-up visit 2	Normal range
Drug therapy	Prednisone 40 mg; darbepoetin 20 ug/week	No prednisone; darbepoetin 50u g/week	Prednisone 5 mg; azathioprine 25 mg; hydroxychloroquine 400 mg; darbepoetin 50 ug/week	
Hemoglobin (g/dl)	8.8	8.3	9.9	13.0-17.0
Erythrocytes (/uL)	3,090	3,290	4,100	4,400-5,900
Leukocytes (/uL)	4,920	2,940	4,370	4,500-11,000
Platelets (/uL)	193,000	203,000	161,000	150,000-450,000
Creatinine (mg/dL)	2.2	2.2	2.4	0.67-1.17
Urea (mg/dL)	101	69	116	16.6-48.5
Amylase (U/L)	166	61	99	13.0-53.0
Lipase (U/L)	115	47	63	13.0-60.0
C3 (g/L)	1.01	0.79	0.71	0.9-1.8
IgG4 (mg/dL)	377	163	109	1-291
Anti-dsDNA antibodies (IU/mL)	200	90	46	<40
Protein-to-creatinine ratio in urine (mg/g)	253	-	105	<200

## Discussion

Overlap syndromes refer to a group of undifferentiated systemic rheumatic diseases in which patients present with features of two or more recognized entities. This is particularly challenging due to the frequent nonspecific serologic abnormalities and the inherent limitations of current classification criteria. Despite the lack of specific evidence, Cervera et al. suggest that at least 15% of patients referred to rheumatology consultations do not fit a single syndrome but instead exhibit overlapping manifestations [6].

IgG4-related disease is a rare condition, and kidney involvement is even rarer. The clinical presentation and histology can vary widely, ranging from typical interstitial nephritis to membranous nephropathy, mesangioproliferative glomerulonephritis, or obstructive uropathy due to retroperitoneal fibrosis [3]. Steroid therapy is the only widely accepted treatment for this disease, although relapses are common, even in the presence of extensive fibrosis [7]. There is no evidence suggesting that a different approach, tailored to the varying histological presentations, would be beneficial. Table 3 presents the diagnostic criteria for both IgG4-related disease and SLE.

**Table 3:** Diagnostic criteria for SLE and IgG4-related disease ANA, antinuclear antibodies; dsDNA, double-stranded DNA; IgG4-RD, immunoglobulin G4-related disease; SLE, systemic lupus erythematosus; SLIC, Systemic Lupus Erythematosus International Collaborating Clinics

Aspect	IgG4-related disease criteria	SLICC 2012 criteria for SLE
Classification requirements	Definite: 1) + 2) + 3); probable: 1) + 3); possible: 1) + 2)	≥4 criteria with at least one clinical and one immunologic OR biopsy-proven lupus nephritis with ANA or anti-dsDNA
Clinical criteria	One or more organs show diffuse or localized swelling or a mass or nodule characteristic of IgG4-RD. In single-organ involvement, lymph node swelling is omitted. The patient had pancreatic involvement.	Cutaneous lupus (acute or chronic), oral ulcers, nonscarring alopecia, synovitis, serositis, renal manifestations, neurologic manifestations, leukopenia, and thrombocytopenia
Serological criteria	Serum IgG4 levels >135 mg/dl	ANA, anti-dsDNA antibodies, anti-Sm antibodies, antiphospholipid antibodies, low complement, and positive direct Coombs test
Histopathological criteria	Positivity for two of the following three criteria: (1) dense lymphocyte and plasma cell infiltration with fibrosis; (2) ratio of IgG4-positive plasma cells /IgG-positive cells greater than 40% and the number of IgG4-positive plasma cells greater than 10 per high-powered field; and (3) typical tissue fibrosis, particularly storiform fibrosis, or obliterative phlebitis. The patient fulfilled all three criteria.	Biopsy-proven lupus nephritis (can be the sole clinical criterion if immunologic criteria are met).

The 2019 American College of Rheumatology/European League Against Rheumatism Classification Criteria for IgG4-Related Disease include positivity for dsDNA antibodies as a serologic exclusion criterion, along with any other disease-specific antibody [5]. However, while these antibodies are highly specific for SLE, they can occasionally be found in other conditions, particularly autoimmune diseases, as well as in otherwise healthy individuals [8]. Interestingly, Yang et al. describe a series of 52 patients in which 12% tested positive for ANA antibodies (regardless of subclass) and 22% for rheumatoid factor, even without overlapping features of other autoimmune diseases, raising questions about the clinical significance of these findings in this context [9]. Furthermore, Hara et al. report no significant differences in the prevalence and patterns of ANA antibodies between IgG4-RD patients and the general population [10]. C3 consumption, which is a hallmark immunologic criterion for SLE diagnosis, can also be a nonspecific finding present in IgG4-related disease and is associated with an increased risk of recurrence [4].

Kidney biopsy is a crucial diagnostic tool in autoimmune diseases with renal involvement. However, its utility is limited by the small sample size, which may not capture disease features if the presentation is focal. It is also important to note that lupus nephritis is not universal in SLE, affecting only about 30-50% of patients [3].

In this case, the patient presented with clinical manifestations that could not be entirely explained by a biopsy-proven IgG4-RD diagnosis. Bone marrow involvement is rare in IgG4-RD, and the biopsy showed only modest IgG4 positivity. The presence of active SLE, without lupus nephritis, provided a reasonable explanation for the persistent cytopenias and C3 consumption, especially after starting steroid treatment and achieving satisfactory kidney function improvement. Voulgarelis et al. further discuss the wide range of histological findings in bone marrow biopsies of SLE patients [11].

Reports of overlapping IgG4-RD and SLE in the literature are scarce. The cases we reviewed all involved patients in their sixth decade of life, and only one showed histological evidence of lupus nephritis [12-14]. This highlights the diversity of clinical manifestations in patients with overlapping syndromes.

In both cases, obtaining histological samples was crucial. Kidney biopsy, when indicated, can significantly influence the diagnostic approach, guiding additional testing, therapeutic management, and surveillance and follow-up plans.

## Conclusions

The challenges in diagnosing these patients may contribute to their underreporting, making it difficult to analyze case series and conduct clinical studies. Available reports suggest that overlapping syndromes may present with varying predominance of the clinical features from each involved entity. Cases like the one presented here indicate that the established exclusion criteria for IgG4-related disease may benefit from rephrasing, encouraging physicians to consider other diagnoses or the possibility of an overlapping syndrome rather than immediately ruling out the disease. A thorough, systematic inquiry into rheumatologic symptoms, along with comprehensive immunologic testing, may help prevent overlooking potential underlying diseases, ensuring a more “complete” treatment approach in a time when evidence to guide management remains limited.
